# Rare genetic variant analysis on blood pressure in related samples

**DOI:** 10.1186/1753-6561-8-S1-S35

**Published:** 2014-06-17

**Authors:** Han Chen, Seung Hoan Choi, Jaeyoung Hong, Chen Lu, Jacqueline N Milton, Catherine Allard, Sean M Lacey, Honghuang Lin, Josée Dupuis

**Affiliations:** 1 Department of Biostatistics, Boston University School of Public Health, 801 Massachusetts Avenue, Boston, MA 02118, USA; 2 Département de Mathématiques, Université de Sherbrooke, 2500 Boulevard de l'Université, Sherbrooke, QC J1K 2R1, Canada; 3 Department of Medicine, Boston University School of Medicine, 72 East Concord Street, Boston, MA 02118, USA

## Abstract

The genetic variants associated with blood pressure identified so far explain only a small proportion of the total heritability of this trait. With recent advances in sequencing technology and statistical methodology, it becomes feasible to study the association between blood pressure and rare genetic variants. Using real baseline phenotype data and imputed dosage data from Genetic Analysis Workshop 18, we performed a candidate gene association analysis. We focused on 8 genes shown to be associated with either systolic or diastolic blood pressure to identify the association with both common and rare genetic variants, and then did a genome-wide rare-variant analysis on blood pressure. We performed association analysis for rare coding and splicing variants within each gene region and all rare variants in each sliding window, using either burden tests or sequence kernel association tests accounting for familial correlation. With a sample size of only 747, we failed to find any novel associated genetic loci. Consequently, we performed analyses on simulated data, with knowledge of the underlying simulating model, to evaluate the type I error rate and power for the methods used in real data analysis.

## Background

Despite the tremendous success of genome-wide association studies (GWAS) to uncover genetic variants influencing complex traits and diseases, only a fraction of the total heritability of these traits is explained by the loci identified so far. Because GWAS focuses on common variants, a possible source of the missing heritability might be rare variants that were not included in the earlier genotyping platforms. The next logical step is to investigate rare variants, an endeavor that is now possible because of the ever-decreasing cost of sequencing.

Whole genome sequencing has the ability to uncover rare variants, but brings its own challenges. Despite a low error rate, the sheer number of base pairs sequenced makes it hard to distinguish very rare mutations from sequencing errors. Moreover, detecting association with rare variants requires very large sample sizes. Several methods to jointly analyze rare variants within a genomic region have been developed, however, and these methods have the potential to pinpoint additional variants contributing to the overall heritability of traits.

Blood pressure (BP) and hypertension are prime examples of the limitations of GWAS. Meta-analysis of GWAS from a large number of cohorts has identified multiple genetic loci over the genome that affect systolic blood pressure (SBP), diastolic blood pressure (DBP), hypertension, or a combination of these traits [1,2]. However, the loci identified to date explain only a small portion of the total heritability in BP.

In this article, we investigate the association of rare variants in genomic regions that have been previously implicated by GWAS to identify the source of the original GWAS signal and to discover additional genetic loci influencing BP using either burden tests adjusting for familial correlation (famBT) or sequence kernel association tests (SKAT) [3] for family samples (famSKAT) [4,5]. We also analyze rare variants genome-wide to uncover additional genomic regions harboring susceptibility variants. Finally, we use the simulated data sets with knowledge of the answer to evaluate type I error and power for famBT and famSKAT in family samples.

## Methods

We used imputed single-nucleotide polymorphism (SNP) dosage files from odd-numbered chromosomes provided by the Genetic Analysis Workshop 18 (GAW18) as our genotypes in all analyses. For real data analysis, we took baseline measurements of the covariates and traits for each participant, defined as the first exam with nonmissing values for age, SBP, DBP, current use of hypertension medications (BPmeds), and current smoking (smoke). We removed participants with at least 1 missing value of these variables in all 4 exams. We also excluded participants on antihypertensive medication at the baseline we defined, resulting in a sample size of 747 participants. Table 1 provides the descriptive information for our subset of participants. Because the distribution of SBP values is highly skewed, rank-normalized SBP (rSBP) values were used in all analyses. DBP values were untransformed. We adjusted for sex, age, and smoking in all our analyses.

**Table 1:** Characteristics of variables of interest at the baseline

N	Sex (F)	Year	Age	SBP	DBP	Smoking
747	57.2%	1992-2005	37 (16-92)	118.7 (80-192)	70.5 (40-114)	22.2%

For continuous variables age, SBP, and DBP, means and ranges are summarized.

For the BP candidate gene study, we performed both common and rare-variant analysis. Common variants were defined as any variants with minor allele frequency (MAF) >5% in our subset of participants, and rare variants were variants with MAF between 0% and 5%. We performed common variant analysis as single-marker association tests using linear mixed-effect models [6] to account for familial correlation and reported the most significant SNP in each region. We performed rare variant analysis for all rare variants within each gene region with famBT and famSKAT [4,5], using Wu weights, which is a beta distribution probability density function of the MAF with parameters 1 and 25 [3]. Both rare-variant approaches are described below.

### Burden tests adjusting for familial correlation (famBT)

Assuming that the sample size is *n, Y* is a vector of the trait of interest; *X *is an *n *by *p *matrix of covariates; *α *is a vector of the covariate effects; *G *is an *n *by *q *matrix of rare variants with columns *G_j_*; *w_j _*is the weight for variant *j*; and this is the combined genotype score:

(1) $$g=\overset{q}{\underset{j=1}{\sum }}w_{j}G_{j}$$

The model is

(2) Y=Xα+gβ+γ+ϵ

where *β *is the effect size for the combined genotype score, *γ *is the random effect vector for familial correlation, and *ε *is the normally distributed error. We assume $\gamma \sim N\left(0,\sigma_{G}^{2}\Phi \right)$, $\epsilon \sim N\left(0,\sigma_{E}^{2}I_{n}\right)$, where $\sigma_{G}^{2}$ and $\sigma_{G}^{2}$ are variance component parameters, and Φ is twice the kinship matrix. The model can be fitted as a linear mixed-effect model and the genotype effect can be tested as *H*_0 _: *β *= 0 versus *H*_1 _: *β *≠ 0 in this framework.

### SKAT for family (famSKAT)

We use the same notation as above, except that *β *is now a vector of length *q*. The model is

(3) Y=Xα+GWβ+γ+ϵ

where *W *is a diagonal matrix of weights *w_j_*, and we assume *β *~*N *(0, *τI_q_*). The genotype effects can be tested as *H*_0 _: *τ*= 0 versus *H*_1 _: *τ*> 0.

For the genome-wide rare-variant analysis on real data, we performed famBT and famSKAT, using both a gene-based coding and splicing variants analysis (GB) and sliding-window analysis (SW). GB was performed for each gene, using only nonsynonymous rare variants and rare variants at the splicing sites. SW was performed for all rare variants in each genomic region of 4000 base pairs (bp) length, with 2000 bp each overlapping with the previous and subsequent windows, regardless of the gene annotation.

### Simulations

In addition to real data analysis, we also performed rare-variant analysis on simulated data sets, with knowledge of the underlying simulating model. To be consistent with the real data analyses, we adjusted for sex, age, and smoking in all analyses, even though simulated smoking is not associated with simulated SBP or DBP. Because we did not have missing data in the simulated data sets, we took the first exam as the baseline and excluded individuals taking antihypertensive medication at baseline. Therefore, the sample size varies slightly in different simulation replicates. We used both famBT and famSKAT for GB and SW, but we analyzed only chromosome 3 because of limited computing resources. We evaluated the type I errors of these approaches using quantitative trait Q1, which was a simulated trait not associated with any genetic variants. We also calculated the empirical power for *MAP4 *on DBP and rSBP.

## Results

### Candidate gene analysis

We chose 8 gene regions (*CASZ1, MTHFR, ULK4, PLEKHA7, CSK, CSK-ULK3, PLCD3, ZNF652*) that were previously reported to be associated with either SBP or DBP [1,2]. Table 2 shows the common variant analysis and rare-variant analysis results. We report the SNPs with the lowest *p *values within each gene. However, the gene-based approach for SKAT and burden test for all associated regions did not reach our threshold for statistical significance (*p *values >0.05/8 = 0.00625). The tests did not identify evidence of association using real data. Candidate gene association results for common-variant analysis on 2 traits, rank normalized SBP and DBP, are also presented in Table 2. There were 3364 common SNPs among these gene regions. None of them was statistically significant after adjusting for multiple testing.

**Table 2:** Candidate gene analysis results

Previous GWAS results							Rare-variant analysis			Common-variant analysis	

**SNP**	**Chr**	**Position**	**Gene**	**Gene position**	**Trait**	***p *value**	**N SNPs**	**famBT** ***p *value**	**famSKAT** ***p *value**	**GWA position**	**GWA *p *value**


rs880315*	1	10796866	*CASZ1*	10696661-10856707	SBP	2.1 × 10^−7^	771	0.563	0.804	10798489	0.0099

rs17367504^§^	1	11862778	*MTHFR*	11845787-11866160	SBP	2.0 × 10^−13^	97	0.665	0.744	11860120	0.0477

rs9815354*	3	41912651	*ULK4*	41288090-42003660	DBP	7.8 × 10^−7^	3104	0.233	0.344	41951111	0.0003

rs381815*	11	16902268	*PLEKHA7*	16809207-17035963	SBP	5.8 × 10^−7^	851	0.262	0.458	16842787	0.0088

rs11024074*	11	16917219	*PLEKHA7*	16809207-17035963	DBP	2.8 × 10^−7^	851	0.995	0.776	17015044	0.0093

rs1378942^§^	15	75078343	*CSK*	75074425-75095539	DBP	1.0 × 10^−23^	91	0.154	0.174	75095157	0.1101

rs6495122*	15	75125645	*CSK-ULK3*	75128459-75135552	DBP	8.0 × 10^−7^	21	0.155	0.712	75130093	0.0709

rs12946454^§^	17	43208121	*PLCD3*	43189008-43209891	SBP	1.0 × 10^−8^	101	0.013	0.490	43202188	0.0449

rs16948048^§^	17	47440466	*ZNF652*	47366568-47439835	DBP	5.0 × 10^−9^	263	0.909	0.611	47411575	0.076

*SNP from CHARGE [2], with *p *value <5.0 × 10^−6^

^§^SNP from GBPGEN [1], with *p *value <5.0 × 10^−8^. Previous GWAS positions were updated to NCBI build 37.

### Genome-wide rare-variant analysis

Table 3 summarizes the genome-wide rare-variant analysis results on the real data. Because there are approximately 20,000 genes in the human genome, we used 2.5 × 10^−6 ^as the genome-wide significance threshold for GB. Given that the human genome has approximately 3 billion bp, we tested approximately 1.5 million sliding windows, each with 4000 bp length and 2000 bp overlap. We thus used 3.3 × 10^−8 ^as the genome-wide significance threshold for SW. However, for both GB and SW, none of the genes or sliding windows was found to be associated with the traits at genome-wide level.

**Table 3:** Genome-wide rare-variant analysis top findings

Gene-based analysis										
	**famBT**					**famSKAT**				

**Trait**	**Gene**	**Chr**	**Position**	**N SNPs**	***p *value**	**Gene**	**Chr**	**Position**	**N SNPs**	***p *value**

DBP	OR2L13	1	248100493-248264224	5	1.8 × 10^−5^	GLIS1	1	53971906-54199877	6	1.1 × 10^−4^

DBP	TRAF1	9	123664671-123691451	2	1.3 × 10^−4^	OR2L13	1	248100493-248264224	5	1.6 × 10^−4^

DBP	TRIM25	17	54965270-54991409	3	4.2 × 10^−4^	DAZL	3	16628299-16647006	3	2.1 × 10^−4^

SBP	KRT14	17	39738531-39743147	4	6.5 × 10^−5^	GTF2H2	5	70330951-70363497	1	8.8 × 10^−5^

SBP	GTF2H2	5	70330951-70363497	1	7.3 × 10^−5^	ERC2	3	55542336-56502391	5	6.9 × 10^−4^

SBP	ACADVL	17	7120444-7128586	8	3.5 × 10^−4^	PCDHB4	5	140501581-140505201	7	1.1 × 10^−3^

Sliding-window analysis										

DBP	CTTNBP2*	7	117615273	14	1.6 × 10^−6^	MIR583*	5	95537956	13	3.8 × 10^−6^

DBP	ITLN2	1	160920490	18	1.9 × 10^−6^	NRCAM	7	107823273	17	7.1 × 10^−6^

DBP	MYO7A	11	76873460	20	3.6 × 10^−6^	INO80	15	41308350	10	1.1 × 10^−5^

SBP	LOC201617*	3	72074162	16	1.6 × 10^−6^	PRKCA	17	64294080	14	3.0 × 10^−6^

SBP	LUC7L3*	17	48832080	16	1.6 × 10^−6^	GUCY1A2*	11	106349460	16	1.9 × 10^−5^

SBP	PSIP1	9	15472910	15	2.6 × 10^−6^	MIR548H3	9	78272910	18	1.6 × 10^−5^

* Indicates nearest gene. For sliding-window analysis, starts of windows are shown as positions; results from windows within 1 Mb of an associated region were removed.

### Simulations

We analyzed all 200 replicates for both GB and SW approaches. Table 4 summarizes the empirical type I errors. For both methods, famBT and famSKAT have correct type I error rates at α levels of 0.05 and 0.001. Table 5 shows empirical power of famBT and famSKAT for the 2 SNP selection approaches. For gene-based analysis, the sample size ranges from 742 to 783, but the number of rare coding variants in *MAP4 *is 6 in all 200 replicates. For each replicate, we performed both famBT and famSKAT on baseline untransformed DBP and rSBP and computed empirical power as the proportion of replicates with *p *values less than the corresponding thresholds. At an α level of 2.5 × 10^−6^, both methods have 100% power to detect association with *MAP4*. However, famBT has a median *p *value of 1.2 × 10^−14 ^for DBP and 3.6 × 10^-16 ^for SBP, whereas famSKAT has a median *p *value of 9.7 × 10^−14 ^for DBP and 1.6 × 10^-15 ^for SBP, suggesting that famBT would be more powerful than famSKAT if a more stringent α level were used. For sliding-window analysis, the sample size also ranges from 742 to 783, and there are 119 windows overlapping with the *MAP4 *gene. The number of rare variants in these windows ranges from 4 to 21. Because the *MAP4 *gene spans a region approximately 239 kb, 60 consecutive windows out of 119 fully cover the gene. For each replicate, we performed both famBT and famSKAT for all 119 windows, selected the smallest *p *value, and multiplied it by 60 for the purpose of adjusting for multiple testing. This adjustment is conservative because the 60 consecutive windows are correlated. Thus, the power of GB and SW may not be directly comparable. However, it is obvious that famSKAT is much more powerful than famBT in SW.

**Table 4:** Empirical type I errors from simulation data sets

	Gene-based analysis			Sliding-window analysis	
			
α Level	famBT	famSKAT		famBT	famSKAT
0.05	0.049	0.048		0.049	0.049
0.001	0.0012	0.0011		0.0010	0.0009

**Table 5:** Empirical power from simulation data sets

		Gene-based analysis				Sliding-window analysis		
				
Trait	Gene	α Level	famBT	famSKAT		α Level	famBT	famSKAT
DBP	*MAP4*	2.5 × 10^−6^	1.0	1.0		3.3 × 10^−8^	0.005	0.815
SBP	*MAP4*	2.5 × 10^−6^	1.0	1.0		3.3 × 10^−8^	0.005	0.945

## Discussion

*MAP4 *encodes microtubule-associated protein 4. This gene is located in chromosome 3p21. The SNPs within the gene region have previously shown a genome-wide association with mean arterial pressure. The top-ranking SNP (rs319690) yields a *p *value of 2.69 × 10^−8 ^[7]. *MAP4 *microtubule decoration restricts with beta-adrenergic receptor recycling, which might explain beta-adrenergic receptor downregulation in heart failure [8].

It is not surprising that in gene-based analysis, famBT is slightly more powerful than famSKAT because, among the 6 rare coding variants in *MAP4*--3_47894286, 3_47913455, 3_47957741, 3_47957996, 3_48040283, and 3_48040284--5 were simulated to be negatively associated with both SBP and DBP. The last SNP, 3_47894286, has perfect linkage disequilibrium (r = 1) with 3_47913455. In such a simulation setting, with SNPs all having the same direction of effect, the burden test should outperform most statistical approaches.

In sliding-window analysis, however, even though *MAP4 *is the gene most significantly associated with both SBP and DBP, some rare regulatory variants were simulated to be positively associated with the traits. As a result, famBT has almost no power to detect the association in this gene region. In contrast, famSKAT performs very well because SKAT allows effects to be in different directions. After adjusting for multiple testing, famSKAT still attains good power even at low α levels.

## Conclusions

The SW method is more computationally intensive than GB because more tests are performed. However, by using SW we can generally test all possible rare variants associated with the trait, no matter where they are located. In many scenarios, intergenic variants, especially those within regulatory regions, also may be associated with quantitative traits. Thus, for rare-variant analysis on real data, unless we have strong a priori knowledge that the associated variants are nonsynonymous, we would recommend running a sliding-window analysis. By using famSKAT, we can perform rare-variant analysis on family data and have much better power than simple burden tests when there are variants with both positive and negative effects.

## Competing interests

The authors declare that they have no competing interests.

## Authors' contributions

HC, HL, and JD designed the overall study; HC, SHC, JH, CL, JNM, and JD conducted statistical analyses and drafted the manuscript; CA, SML, and HL performed data quality control and gene annotation. All authors read and approved the final manuscript.
